# Immersive Virtual Reality: A Novel Approach to Second Language Vocabulary Acquisition in K-12 Education

**DOI:** 10.3390/s24227185

**Published:** 2024-11-09

**Authors:** Mohammed Alfadil

**Affiliations:** Department of Educational Technology, School of Education and Behavioral Sciences, University of Northern Colorado, Greeley, CO 80639, USA; alfadil1777@gmail.com

**Keywords:** immersive virtual reality game, immersive virtual environment, vocabulary acquisition, second language, UTAUT model

## Abstract

Today, immersive virtual reality (IVR) is increasing in popularity in a broad range of fields, including science, pedagogy, engineering and so forth. Therefore, this study discusses the Unified Theory of Acceptance and Use of Technology (UTAUT), which can be used to examine the factors that influence the adoption of immersive VR in the classroom, particularly in second language (L2) vocabulary acquisition. The sample for this study included 32 intermediate students and their teacher. For the purpose of evaluation, the researcher used partial least squares structural equation modelling (PLS-SEM) techniques to analyze the results. The findings of the students’ survey showed that performance expectancy, effort expectancy and social influence were seen to have had a positive impact on the intention to use immersive VR. Likewise, the findings indicated that facilitating conditions were seen to have had a positive impact on the use behavior of actually using immersive VR, whereas behavioral intentions did not. In addition, the teacher’s survey demonstrated a favorable view regarding the potential of immersive VR technology to support teaching L2 vocabulary acquisition. This particular study encouraged educators and educational technologists to utilize immersive VR games as a teaching–learning tool to reduce the challenge of L2 vocabulary acquisition.

## 1. Introduction

If not now, immersive virtual reality (IVR) will soon become the future of the coming generations and will take its place in both adult education and workplace development. Huawei Global Industry Vision (GIV) predicts that by 2025, more than 337 million people and 10% of companies will use VR technology. Over the next decade, VR is going to be a very promising market through mixing artificial intelligence (AI), augmented reality (AR), 5G and VR to open up new vistas for people, business, culture, education and training, and will generate more than USD 1.3 trillion in revenue [1]. This supports the belief that VR is the core technology for learning spaces and innovative cultures.

Virtual reality is a promising new technology that provides the illusion of immersion in an environment that is digitally generated and does not physically exist [2,3]. Jennifer Wu and Philipp Kraemer [4] report that “the development and implementation of the Tactical Language Training System successfully utilizes a virtual environment to promote foreign language learning among U.S military personnel. Learners practice interactions with virtual native speakers in a mission-based game” [5].

Recent research on AR with regard to teaching science vocabulary [6] confirmed that the use of AR is an effective way to teach science vocabulary to college students with intellectual disabilities and autism. One student with autism significantly improved their understanding of human bone vocabulary, from 30% to 80% accuracy, after using AR. In the same study, all participants expressed a strong preference for AR learning and believed that it would enhance their science vocabulary. Because augmented reality and virtual reality technologies share many similarities, it is possible that VR could also be a valuable tool for teaching language vocabulary to students.

The research literature on using VR technology to teach L2 vocabulary acquisition to students is limited. There is a significant gap in the literature regarding the effectiveness of depth of immersion, the extent of engagement with the virtual environment and the quality of VR environment design. Existing research has not thoroughly explored these factors and their impact on learning achievements. Therefore, this study intends to help readers gain insight into the factors that impact the intentions of students and teachers’ adoption of immersive VR in classrooms, especially in L2 vocabulary acquisition. The significance of this study rests upon how a fully immersive VR environment mode enhances students’ learning experiences and improves second language vocabulary acquisition. This particular study encouraged educators and educational technologists to utilize immersive VR games as a teaching–learning tool to reduce the challenge of L2 vocabulary acquisition. It seems reasonable to assume that the Unified Theory of Acceptance and Use of Technology (UTAUT) model could be used to study the acceptance and use of immersive VR technology in the classroom, particularly in L2 vocabulary acquisition. Therefore, the researcher introduced subjective task value to the UTAUT to address the research questions.

To define the study’s purpose, the researcher formulated the following questions:

Q1: What are the factors that influence students’ intentions to use immersive virtual reality technology as a learning method for vocabulary acquisition?

Q2: What is the perception of a teacher who integrates immersive virtual reality technology into his pedagogical practices for teaching vocabulary acquisition to intermediate school students?

### 1.1. Learning Vocabulary

A strong vocabulary is highly valued in a portfolio, but a significant effort is required to build a large and varied word bank. A study that measured vocabulary size (number of known words) revealed that students’ accumulated reading vocabulary was roughly 25,000 words by the end of primary school and 50,000 words by high school [7]. Improving the vocabulary of K-12 pupils has been challenging. Traditional teaching methods often rely on boring memorization, citation and repetition. However, new approaches focus on making learning fun and personalized for each student [8,9].

Traditionally, it has been a common preconception that schools are the primary places where vocabulary improves. However, Brahic [10] challenged this idea. The author points out that schools are a relatively recent invention in the field of human history. For centuries, people have learned without formal classrooms, suggesting that vocabulary growth can occur outside of educational settings.

How do humans learn a language without school? A renowned psychologist provides the answer that learning happens best through real-life experiences, interacting with others and watching what others do. Research by Hunt [11] concluded that online platforms such as YouTube show how technology can help people learn new skills in ways that were impossible before. For example, someone wanting to play a guitar like Brian Setzer can watch his videos “Stray Cats” and learn from him. However, this kind of learning is mostly one-way, without direct interaction

In the past, car owners were able to fill up the gas tank and drive the car without understanding the internal mechanics of the engine. However, the advent of technology has empowered individuals to access detailed information and tutorials on vehicle engines through platforms such as YouTube [11]. While this form of independent learning has enabled individuals to acquire foundational vocabulary related to their chosen subject matter, it may not provide sufficient depth for an advanced understanding or expertise.

In this paper, the term “second language” encompasses any language learned subsequent to one’s native tongue. For native English speakers, this might include Spanish, Thai, Filipino, Mandarin or countless other languages [12]. Second language acquisition therefore refers to the process by which individuals learn an additional language. It is crucial to distinguish this process from language pedagogy, as the former focuses on the learner’s experience rather than on the teaching methodologies employed [13,14].

### 1.2. Virtual Reality

Virtual reality can be defined as a computer-generated, three-dimensional environment that can be explored and interacted with by individuals through specialized electronic equipment, such as head-mounted displays or sensor-equipped gloves [15,16]. Virtual environments constitute a foundational component of diverse technological applications including virtual reality, mixed reality, augmented reality and 3D video games. An immersive virtual reality environment (IVRE) can be defined as a 360-degree virtual environment system that permits users to move freely and manipulate and interact with objects seamlessly, as in real-life situations, by turning their head and body [4,17,18].

### 1.3. Immersive Virtual Reality Environment

**Figure 1:**
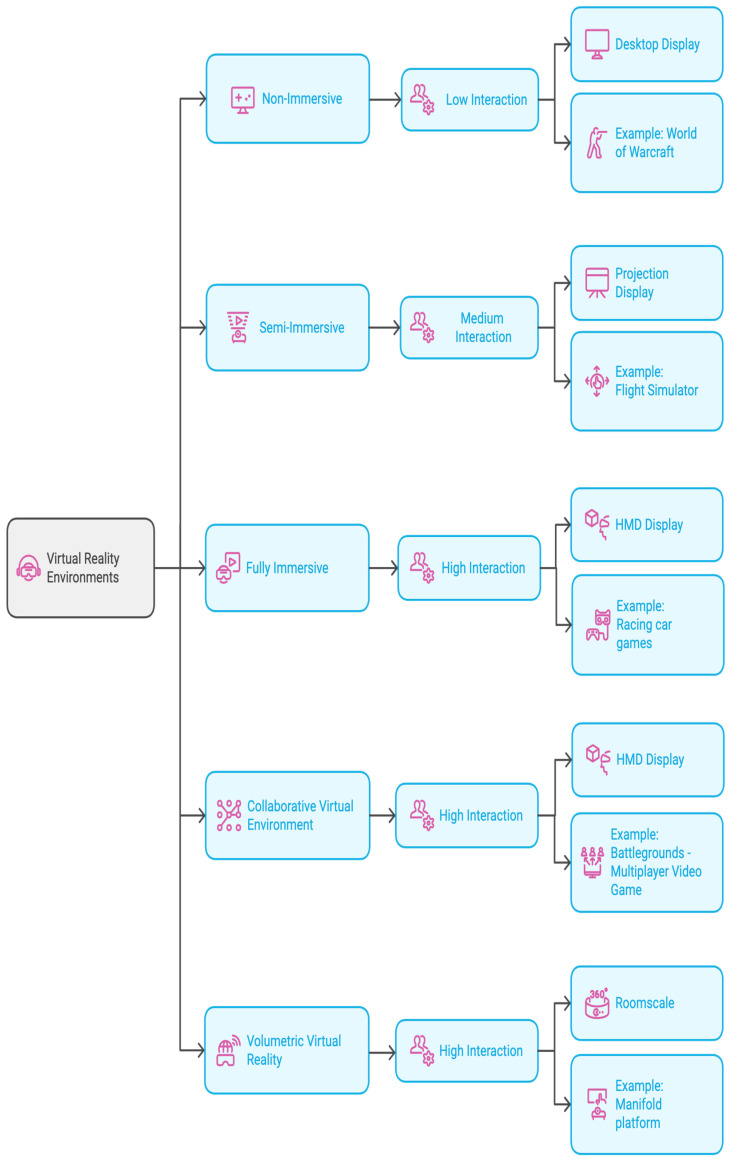
Summary of virtual reality environment types based on level of immersion and features.

The rapid progress in the field of VR is linked to the user’s immersion level in the VR environment, which is well-known as an immersive virtual reality environment [19]. Thus, there are many types of VR environments in terms of their level of immersion and features. For an overview and comparison of the environments, see Figure 1. In this figure, the VR environments were rated according to the level of immersion, level of interaction and display device(s) used, where three-dimensional space on a desktop was rated as non-immersion and low interaction, a concave screen simulator was rated as medium immersion and medium interaction, and VR head-mounted display and VR glasses were rated as high immersion and high interaction. For example, a collaborative virtual environment is a shared virtual environment in which several people from various locations can interact with each other to share ideas and experiences, accomplish tasks or play games [20]. The collaborative virtual environment can be seen clearly in a video game called Players Unknown Battle-Ground (PUBG), in which many players interact with each other through headsets, microphones and chatting.

However, collaborative virtual environments have recently begun to attract the attention of researchers in various fields, such as education and training to support distance learning [21,22,23]; healthcare in phobia treatment [24]; medicine in surgery simulation [25,26]; and marketing to increase consumer interaction [27].

The current study adopted a fully immersive VR environment in which users interact with it through using head-mounted display (HMD) buttons and head and body movements. There is increasing growth in applications based on immersive VR environments that can be seen in virtual shooter gaming zones and racing car games, as shown in Figure 1. As virtual reality technology becomes more ubiquitous and immersive, new possibilities for teaching and learning provided by immersive virtual reality have been increasingly recognized by educational researchers. This has introduced a new dimension to education and offers opportunities for learning experiences and teaching processes by generating an interactive and immersive environment [28,29]. We must pay attention to ensure that immersive VR is well structured, so it will enhance students’ learning; if it is not well structured, implementing immersive VR in the learning process will not be helpful. If immersive VR is well-designed and implemented effectively, students can be motivated, engaged and enthusiastic about learning [30].

### 1.4. Efficiency of the Immersive VR Application

Many people have traditionally used immersive VR technology only for entertainment purposes, ignoring the critical role it may play in providing an active learning environment. Lan et al. [31] examined how different learning contexts can impact L2 vocabulary learning by comparing picture–word association vocabulary learning versus learning via an online virtual environment platform. The results showed that the participants in the virtual environment group had a faster learning trajectory than the participants in the picture–word group. This finding suggests that simulated embodied experiences in a virtual environment play a critical role in enhancing L2 vocabulary learning.

The research literature has investigated the use of immersive virtual environments to access the environment that language students can experience without leaving their current location. In this sense, Si [32] utilized an immersive environment through using a desktop computer game and Kinect motion sensors to help learners by promoting embodied experiences and improved communication skills. From an educational and sustainable point of view, a large proportion of studies have promoted the use of VR environments in L2 language instruction to improve reading and listening skills [33,34], and to promote collaboration and L2 communication skills [35,36]. These studies found that a simulated embodied experience in a virtual environment facilitated participants’ L2 performance and engagement.

## 2. Theoretical Perspective and Research Hypotheses

In this current work, the researcher focused on identifying the factors that affect students’ adoption of immersive VR games in English as a Second Language (ESL) vocabulary acquisition to assess whether immersive VR games may serve as a promising approach for facilitating L2 vocabulary acquisition. The researcher selected L2 language vocabulary acquisition for this study because of the limited number of studies conducted on L2 vocabulary acquisition through immersive VR technology and the availability of immersive VR software on the topic.

The Unified Theory of Acceptance and Use of Technology (UTAUT) model is well suited to the context of this study. Venkatesh et al. [37] employed the UTAUT model to gain insights into the acceptance behavior of individuals across range of fields. This model constantly evolves through the incorporation of new factors and interrelationships [38]. For instance, the inclusion of the IT Capability factor [39,40] and self-efficacy perceptions [41,42,43] has contributed to a more comprehensive understanding of the acceptance and utilization of innovative technologies and methodologies. Despite the limitations associated with the UTAUT model, numerous studies have confirmed its effectiveness of the UTAUT model in evaluating technology acceptance in educational contexts across various settings, such as the assessment of interactive whiteboard usage [44,45], virtual classrooms [46,47], MOOCs [48,49] and learning platforms [50,51,52,53]. This study adopts the aforementioned model, which incorporates the following determinants [37] (pp. 447–453):

1. Performance Expectancy (PE) is described as “the degree to which the user expects that using the system will help him/her attain gains in job performance”.
2. Social Influence (SI) is “the degree to which an individual perceives that important others believe he or she should use the new system”.
3. Effort Expectancy (EE) is defined as “the degree of ease associated with the use of the system”.
4. Facilitating Conditions (FC) refers to “the degree to which an individual believes that an organisational and technical infrastructure exists to support the use of the system”.

**Figure 2:**
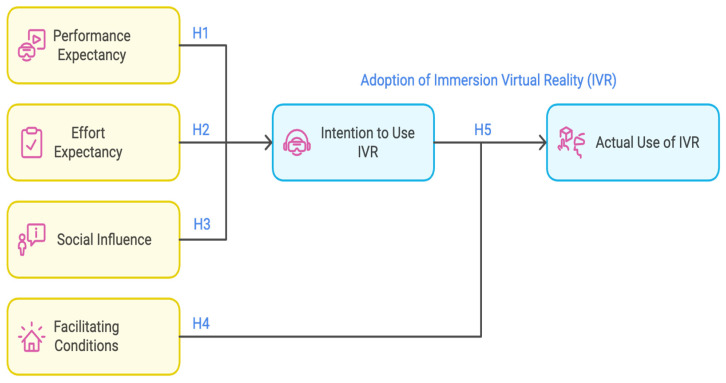
A modified version of the UTAUT model for understanding factors that are affecting adoption of VR.

As can be seen in Figure 2, the UTAUT model posits three key determinants that directly influence the intention to use (performance expectancy, effort expectancy and social influence) and two key determinants that directly influence actual use (intention to use and facilitating conditions). This study did not consider the moderating effects of gender, age, experience or selection method (convenience sampling). Since all the participants were intermediate school students, their gender, age, experience and selection method were similar. Therefore, the researcher made certain adjustments to the research model (see Figure 2).

### Research Hypotheses

Based on the UTAUT, the researcher formulated and tested the following Hypotheses to elucidate the study’s aims and objectives:

**Hypothesis** **1** *($H_{1})$: Performance expectancy positively influences the behavioral intentions to use immersive VR;*

**Hypothesis** **2** *($H_{2})$: Effort expectancy positively influences the behavioral intentions to use immersive VR;*

**Hypothesis** **3** *($H_{3})$: Social influence positively influences the behavioral intention to use immersive VR;*

**Hypothesis** **4** *($H_{4})$: Facilitating condition of immersive VR positively influences the use behavior of actually using immersive VR;*

**Hypothesis** **5** *($H_{5})$: Behavioral intentions to use immersive VR positively influence the use behavior of actually using immersive VR.*

## 3. Methods

**Figure 3:**
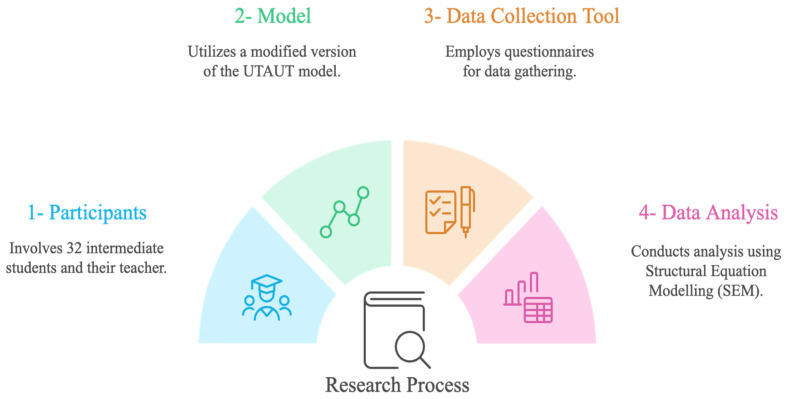
Research process.

This study used UTAUT to explore the factors that influence the adoption of immersive VR in pedagogical practices for learning L2 vocabulary acquisition (see Figure 3). Moreover, this study used a survey research approach to investigate teacher’ perception of the effectiveness of integrating an immersive VR vocabulary learning game into his pedagogical practices for teaching L2 vocabulary acquisition to intermediate school students. The intent of the teacher’s survey was to support the investigation of students’ perceptions towards accepting and using immersive VR to support L2 vocabulary acquisition.

### 3.1. Sample

**Figure 4:**
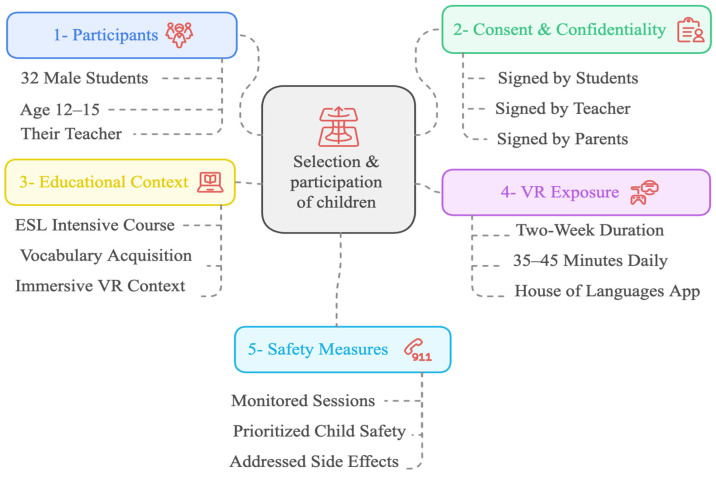
Selection and participation of children.

The sample included a total of s male students (aged 12 to 15, M = 14.02, SD = 0.47) and their teacher, who were engaged in non-native language vocabulary acquisition in the immersive VR context in the eastern region of Saudi Arabia. The educational system there is segregated by gender, which means that males are separated from females in the place of learning. Participants were non-randomly selected using convenience sampling. Convenience sampling is a non-probability sampling technique that involves selecting participants based on their accessibility and availability to researchers [54]. The participants were chosen based on those enrolled in an ESL-intensive course in an immersive VR context for two weeks. The teacher had been a teacher for six years. The teacher used immersive VR technology each day during the two weeks, totaling 35–45 min daily of exposure to immersive VR. The consent forms and confidentiality statements were signed by the students, teacher and parents (see Figure 4).

### 3.2. Instruments

#### 3.2.1. Perception Surveys

Perception surveys serve as value-chain assessment tools to elicit teacher and student perspectives on the efficacy of immersive VR technology. The researcher constructed two distinct surveys and validated them by experts and the classroom teacher to measure the perceptions of both the students, using an immersive VR House of Languages game as a learning resource and teacher perceptions of the game’s integration into the instructional process.

The students’ surveys measured their usage and acceptance of the immersive VR game within the learning context. The students’ survey consisted of two sections: (1) the demographics of each of the participants and (2) the UTAUT section, which consisted of six categories: performance expectancy, effort expectancy, social influence, facilitating conditions, behavioral intention and the actual use of immersive VR. All UTAUT categories were measured using the items listed in Table 1, which were derived from the original UTAUT model [37,55] and adapted from previous studies applying UTAUT to specific contexts, such as mobile learning [56], E-Learning Technologies [57] and E-Learning Acceptance and Effectiveness [58].

The teacher survey measured teacher’s perceptions of the usefulness of immersive VR game in the learning process. The surveys consisted of two sections: (1) the demographics of each participant and (2) teacher’s perceptions of the effectiveness of immersive VR. The teacher’s perception of immersive VR consisted of four items in terms of usefulness, feasibility, effectiveness in teaching and whether it addressed individual learners’ differences in one learning activity. Responses to all survey items were captured using a 5-point Likert scale ranging from “1 = strongly disagree” to “5 = strongly agree”. Survey completion time was estimated at five to ten minutes via email delivery.

#### 3.2.2. House of Languages Immersive Virtual Reality App

This study proposes the integration of an immersive virtual reality game, “House of Languages”, as a pedagogical tool for vocabulary acquisition. House of Languages, created by the Estonian game company Fox3D, is a VR application designed to facilitate language learning in Spanish, German, Russian and English [60]. The “House of Languages” VR game immerses players in a cartoon-like environment guided by a raccoon character named Mr. Woo. As players explore various 3D objects within the game’s 12 distinct locations (e.g., airport, cinema, zoo, cafe, school and museum), they are exposed to object names and corresponding pronunciations. The progressive difficulty level of the game, beginning with object identification and culminating in word-guessing and puzzle-based mini-games, aims to enhance vocabulary acquisition and language skills. This study utilized Samsung VR headsets and smartphones in the experimental phase. It is important to note that the “House of Languages” is exclusively compatible with the Samsung Gear VR devices.

### 3.3. Procedures

**Figure 5:**
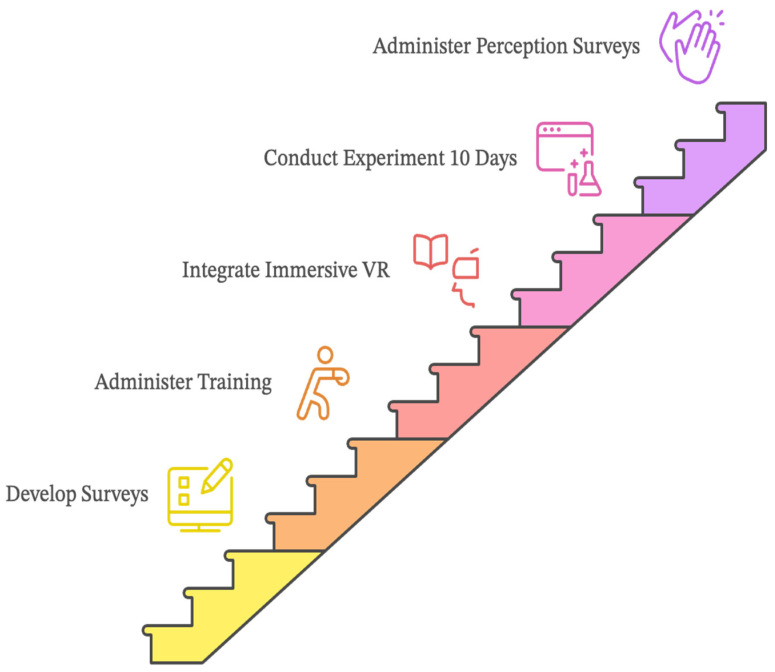
Procedural steps and timeline.

Research design constitutes a comprehensive framework for testing a specific hypothesis or research question [61]. In this study, the researcher believes that the study required a survey design to provide a clear path to understanding the research hypotheses. As can be seen from Figure 5, the experiment lasted for 12 days and was performed four times per week, commencing and concluding with an evaluation Perception Survey. There were two separate surveys were conducted to measure the perceptions of both students, using the immersive VR House of Languages as a learning tool, and the teacher, regarding the implementation of the immersive virtual reality game in the learning and teaching process. 

Prior to the experiment, participants were provided with an introductory video and a thirty-minute training session to familiarize them with the VR gear, the House of Languages app and the overall procedure. The training focused on the VR headset operation, hand-movement controls and game setup to ensure participant comfort and familiarity with the technology.

The students depended only on the immersive VR game during the experimental period to meet the course requirements. The teacher used immersive VR technology each day for 12 days, totaling 35–45 min daily of exposure to immersive VR. The students exchanged VR headsets after participating in an immersive VR game for an established, timed session to ensure that all students had the same opportunity to play the game in a controlled environment. The actual learning time differed from one student to another but ranged between five and eight minutes in each trial based on individual achievement in each task. Those who were waiting for their experience were monitored by the teacher. The perception surveys (both teacher’s and students’) were collected in the last class session of the experimental period and were processed and analyzed quantitatively.

## 4. The Analysis Techniques

For the purpose of evaluation, the researcher used Partial Least Squares-Structural Equation Modeling (PLS-SEM) analysis techniques to analyze the students’ perceptions of the factors that influence the adoption of immersive VR technology in the classroom. Two types of evaluation were conducted: an outer evaluation model (measurement model) and an inner evaluation model (structural model). In the outer model evaluation, reliability and validity were assessed by examining indicator reliability, internal consistency reliability, convergent validity and discriminant validity. For inner model evaluation, the structural model and hypotheses were tested to confirm the research hypotheses.

## 5. Results

### 5.1. UTAUT Questionnaire

After analyzing the data collected from the participants’ UTAUT survey, the following results were obtained.

#### 5.1.1. Outer Model Evaluation

Assess Indicator Reliability. The reliability of the indicators was assessed as part of the outer model evaluation in PLS-SEM analysis. This encompassed the examination of individual indicator reliability, internal consistency, convergent validity and discriminant validity. According to the findings of Hair et al. [62], reliability indicators are considered acceptable when the standardized indicator loadings are greater than or equal to 0.70. In Table 2, the outer loadings of all individual indicators are shown to be above 0.7. Thus, the reliability indicator is confirmed to be reliable.

Assess Internal Consistency Reliability. According to Hair et al. [62], the measurement of internal consistency reliability should not rely on the Cronbach’s alpha. They indicated that Cronbach’s alpha is a less precise measure of reliability as the items are unweighted. Instead, they proposed using composite reliability ${rho}_{c}$ as the preferred measure and it should be >0.70. Upon examining Table 3, it can be concluded that the composite reliability of all variables in this study is deemed acceptable.

Assess the Convergent Validity. Convergent validity was estimated to evaluate construct validity of the measurement model. Hair et al. [62], suggested that convergent validity measurement uses the average variance extracted (AVE), where the AVE value should be ≥0.50. As Table 3 shows, the AVE values for each construct exceeded 0.50. Thus, based on these findings, it is concluded that the convergent validity of all variables is deemed acceptable.

Assess Discriminant Validity. Last, discriminant validity was measured using the Fronell–Larcker criterion. Discriminant validity refers to the degree to which a construct is empirically distinct from other constructs in a structural model [63,64]. As presented in Table 4, all the constructs were distinct among each other and less than the suggested value of 0.85 [65]. Therefore, the discriminant validity was accepted.

#### 5.1.2. Inner Model Evaluation

**Figure 6:**
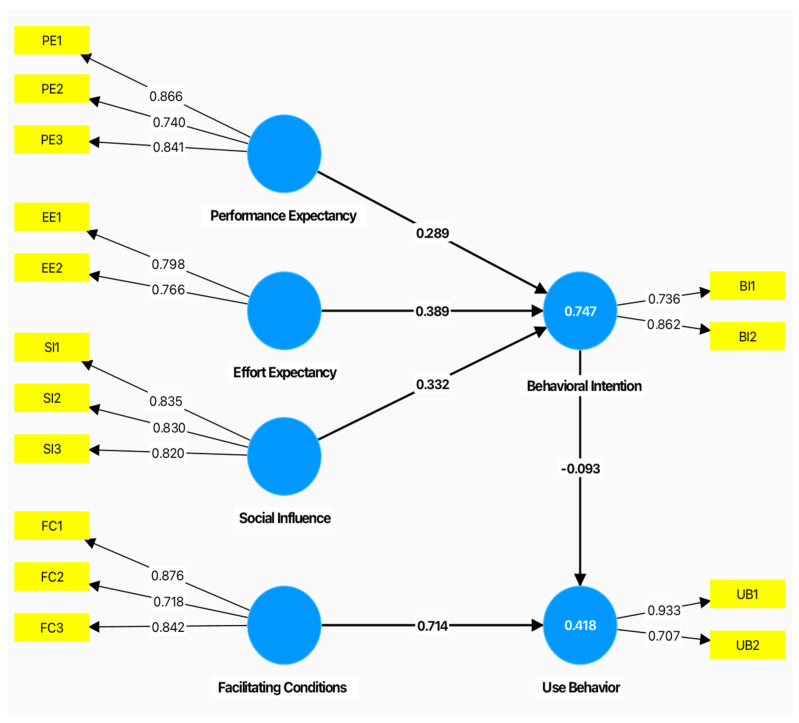
Structural model. Arrows toward the yellow box indicate outer loadings, while arrows pointing to the blue circle represent standardize coefficient effect. R2 is shown inside the blue circle.

Structural Model. Testing the structural model was the next step. Figure 6 shows the final structural model that results from implementing the refinement criteria mentioned in the measurement model section, which contains 15 items. The R Square ($R^{2})$ explains the variance in the dependent variables explained by the independent variables and is therefore a measure of the model’s explanatory power. As a point of reference, $R^{2}$ values of 0.75, 0.50 and 0.25 can be categorized as substantial, moderate and weak, respectively [66,67]. Based on the $R^{2}$ results obtained using SmartPLS software (version 4.1.0.8), this model explained approximately 74.7% of the variance in behavioral intentions and 41.8% of the variance in usage behavior (See Figure 6). Since all the values of $R^{2}$ are within the acceptable range, this model is reasonably consistent with the data.

Hypotheses Testing. The next step was to test the hypothesized relationships between UTAUT factors, behavioral intentions and use behavior of actually using immersive VR in second language (L2) vocabulary acquisition. For the inner model, the t-statistic should exceed 1.96 and the *p*-value should be less than 0.05 to confirm the hypotheses [62,68]. Table 5 presents a summary of the supported and non-supported hypotheses as following:

$H_{1}$ evaluates whether performance expectancy positively affects behavioral intention to use immersive VR. The results revealed that performance expectancy had a positive impact on students’ behavioral intention to use immersive VR (β = 0.289, t = 1.976, *p* = 0.049 < 0.05). Hence, $H_{1}$ was supported.

$H_{2}$ evaluates whether effort expectancy positively affects behavioral intention to use immersive VR. The results revealed that effort expectancy had a positive impact on students’ behavioral intention to use immersive VR (β = 0.389, t = 2.375, *p* = 0.018 < 0.05). Hence, $H_{2}$ was supported.

$H_{3}$ evaluates whether social influence positively affects behavioral intention to use immersive VR. The results revealed that social influence had a positive impact on students’ behavioral intention to use immersive VR (β = 0.332, t = 2.053, *p* = 0.041 < 0.05). Hence, $H_{3}$ was supported.

$H_{4}$ evaluates whether facilitating conditions positively affect the use behavior of actually using immersive VR. The results revealed that the facilitating conditions had a positive impact on students’ use behavior of actually using immersive VR (β = 0.714, t = 2.870, *p* = 0.004 < 0.05). Hence, $H_{4}$ was supported.

$H_{5}$ evaluates whether behavioral intention positively affects the use behavior of actually using immersive VR. The results revealed that behavioral intention had a positive impact on students’ use behavior of actually using immersive VR (β = −0.093, t = 0.415, *p* = 0.679 > 0.05). Hence, $H_{5}$ was not supported.

### 5.2. Teacher’s Survey

**Figure 7:**
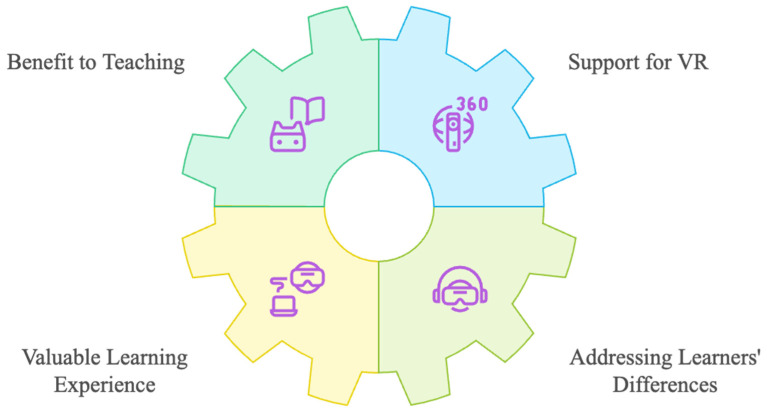
Teacher perception of the effectiveness of VR.

As only a single survey response was collected, a reliability assessment was not feasible. The results from the teacher’s survey revealed overall high levels of confidence with the usefulness of the immersive VR game play experience and welcomed this technology in terms of the learning and teaching process. As can be seen from Figure 7, the teacher indicated that he strongly agreed that using the immersive VR game in class benefited his teaching (Item 1). Moreover, the teacher exhibited unequivocal support for both immersive VR technology and the “House of Languages” VR game within the classroom context (Item 2). The teacher perceived the immersive VR game as a valuable learning experience (Item 3), and strongly endorsed its capacity to address individual learners’ differences in one learning activity (Item 4).

## 6. Discussion

The study applied the UTAUT model to examine the factors that influence the adoption of immersive VR technology in the field of 2 L vocabulary acquisition. Overall, the findings indicate substantial acceptance of immersive VR technology among both students and the teacher, as all but one of the path coefficients were statistically significant.

### 6.1. Performance Expectancy (PE)

Students’ perceptions regarding the efficacy of the immersive VRG House of Languages as a vocabulary-acquisition tool provided further positive results. Students reported that the VRG House of Languages enhanced classroom engagement, increased self-confidence and felt more challenged by the instruction that allowed them to take virtual field trips over different topics. These positive perceptions demonstrate at least a cognitive difference between the two pedagogical approaches (immersive VR and traditional) as a result of the successful integration of immersive VR into the learning process. This finding aligns with those from previous studies using different kinds of sampling methods and measurement approaches to the key variables [69]. The implementation of a 3D immersive virtual environment within a physical classroom setting fostered heightened levels of confidence, openness, participation, creativity and comprehension among learners, suggesting strong intrinsic motivation to engage with the learning process. Another explanation might be that the novelty and excitement of using immersive VR may have contributed to the increased jump in students’ positive responses, especially since most students do not use immersive virtual reality headsets in their daily life.

### 6.2. Effort Expectancy (EE)

In this study, there was no particular concern about handling VR or any intolerance of the immersive VRG House of Languages application noted in the students’ perception surveys. One potential explanation for this high level of acceptance is students’ familiarity with similar devices in their personal lives. Their prior experience with technology, particularly within their home environment in recent years, has facilitated rapid and comfortable adaptation to new equipment [70,71,72]. The students’ capacity for independent vocabulary acquisition using the House of Languages immersive VR game further suggests that immersive VR technology is a valuable tool beyond school doors.

### 6.3. Social Influences (SI)

Throughout the experimental phase, the participants expressed a desire to utilize immersive VR technology to learn additional content. The majority of students stated that their school should continue to use immersive VR technology to learn other content. This finding is very similar to those of other studies conducted to obtain students’ perceptions of immersive technologies in the learning process [73,74,75,76,77]. This might be attributed to immersive VR technology’s capacity to create highly realistic and engaging learning environments.

### 6.4. Facilitating Conditions (FC)

Additionally, the opportunity to interact within an immersive VR environment with a native English-speaking virtual teacher (Mr. Woo—raccoon character host) may have facilitated superior productive vocabulary acquisition compared to interacting with a real, but non-native, English-speaking classroom teacher. Moreover, students showed a high level of satisfaction as they all interacted with their virtual teacher within immersive virtual environments at the same time, which can rarely happen in conventional education.

These findings align with a recent study in the same domain [78,79] which also supported demonstrated the efficacy of VR for vocabulary learning. These findings provide empirical support for the hypothesis that liking the program would enhance students’ learning in content topics. This leads to the conclusion that increased exposure to immersive VR technology may correlate with different learning outcomes compared to traditional educational methods.

### 6.5. Behavioral Intentions (BI)

Although previous studies have suggested that behavior intention has a significant impact on actual use [80,81,82,83], this study found that behavioral intention does not significantly affect actual use immersive VR. A possible cause of the insignificant impact of behavior intention on actual usage could be the potential disappearance or stability of the intention over time due to unforeseen obstacles to action [84]. Consequently, measuring behavior intention and actual using at various stages throughout the implementation process may yield different results. In addition, the limited number of questions related to behavioral intention in this study seems to be a possible explanation for this discrepancy.

### 6.6. Teacher Loved Immersive Virtual Reality Technology

The teacher had a positive perception of using the immersive VRG House of Languages as a vocabulary-acquisition tool, as indicated by uniformly strong agreement across all survey items. This positive outlook may be attributed to the teacher’s personality and general preference for technological advancement and prior experience with similar technologies. However, it is essential to acknowledge that factors such as age and ability to cope with new technology can influence teacher perceptions of VR integration. Older teachers with limited technological exposure may require additional support to effectively incorporate VR into their teaching practice.

The teacher strongly agreed that the immersive VR game allows teachers to address individual learner needs within a single activity, potentially due to the increased instructional time for lower-achieving vocabulary students. This finding is consistent with the results of similar studies, which found that the VR environment offers promise in accommodating individual differences pertaining to learning styles [85,86,87]. Teacher responses indicated that immersive VR was more effective than traditional methods in enhancing students’ vocabulary acquisition. This may be attributed to the teacher’s perception of VR as a novel and engaging pedagogical tool compared to previously employed teaching tools. Another possible explanation is the limited technological integration of vocabulary instruction [88,89]. Rather than substituting existing technologies, the integration of immersive VR has effectively demonstrated its capacity to enhance students’ vocabulary acquisition.

Overall, teacher acceptance of immersive VR as an effective pedagogical approach aligns with the hypothesis that it could potentially reduce student distractibility, although this was not directly investigated in this study. In light of the above, the House of Languages VR game provides a practical path for teachers to integrate immersive VR into their classrooms. These findings underscore the need to promote VR technology adoption among educators who may be unaware of the potential to enhance students’ learning experiences.

## 7. Limitations and Future Research Directions

This study has some limitations. Firstly, the small sample size of the teacher survey (*n* = 1) restricted the generalizability of the findings and precluded a comprehensive understanding of immersive VR use in ESL vocabulary acquisition. Moreover, the participants were selected using a convenience sample, rather than a random sample. Additionally, the study’s exclusive focus on male participants limited its applicability to a wider population. The educational system in which this study was conducted (Saudi Arabia) is segregated by gender, meaning that males are separated from females in the place of learning. As a result, the study results cannot easily be generalized to all genders.

Future research should consider replicating this study with a larger sample size and consider additional variables. Further work will consider the research design and different languages (Spanish, French, German, Arabic, etc.) to determine whether the results can be generalized across different languages and different research designs. An additional consideration for further work will be different age groups, such as adults or older populations, who might provide different results.

## 8. Implications on Learning Process, Students and Society

Despite the challenges associated with integrating immersive VR into education, it holds immense potential to shape future learning environments and equip learners for the workforce. Furthermore, immersive VR can significantly enhance student engagement in the learning process by providing interactive and immersive experience. The integration of immersive VR can be tailored to individual learners’ needs, allowing personalized learning experiences. Additionally, immersive VR can facilitate collaborative learning experiences by enabling students to interact with each other in virtual environments. The adoption of VR in education can drive innovation and lead to the development of new teaching and learning methods. Culturally, immersive VR can expose students to different cultures and perspectives, thereby promoting their cultural understanding.

## 9. Conclusions

The primary objective of this study was to examine the factors that influence the adoption of immersive VR technology in the learning process, particularly in ESL vocabulary acquisition. The findings from both participant and teacher surveys were positive and supported the integration of immersive virtual reality as a pedagogical tool for second language vocabulary acquisition. Students indicated that immersive VR technology made the learning process interesting and challenging and allowed them to discover different topics and environments in one learning set, which could not happen with the traditional learning method. The teacher also welcomed immersive VR technology as a promising technique to address individual learners’ differences in second language vocabulary acquisition.

This study contributes to the body of knowledge in two ways. First, it is an essential step in understanding the role of immersive VR as a promising technology for reducing the challenges in L2 vocabulary acquisition, as many educators have not yet comprehended the effectiveness of adopting such immersive technology in their educational practices to enhance students’ learning experiences. Second, this study is an essential starting point for further research to address different second language aspects, e.g., memorizing, pronouncing, grammar, writing, reading and spelling. 

## Figures and Tables

**Table 1:** Students’ survey items.

Variable	Survey Items	References
Performance Expectancy (PE)	PE1: Using immersive VR game *House of Languages* improves my vocabulary acquisition. PE2: I would find immersive VR useful in my school study. PE3: Using immersive VR made the classroom more interesting and felt more confident and challenged with the virtual field trips.	[37]
Effort Expectancy (EE)	EE1: I would find immersive VR game *House of Languages* is easy for me to use. EE2: My learning activities with immersive VR game *House of Languages* are clear and understandable.	[37]
Social Influences (SI)	SI1: My peers and teachers think that I should use immersive VR to learn other content. SI2: People who are important to me think that I should use immersive VR. SI3: People who affect my learning behavior think that I should use immersive VR.	[37]
Facilitating Conditions (FC)	FC1: I have the resources necessary to use immersive VR. FC2: I have the knowledge necessary to use immersive VR. FC3: If I have problems using immersive VR game *House of Languages*, I could solve them very quickly.	[37]
Behavioral Intentions (BI)	BI1: I intend to use immersive VR in my future learning activities. BI2: I would use immersive VR game to improve my L2 vocabulary acquisition.	[37]
Use Behavior (UB)	UB1: I consider myself a regular user of immersive VR. UB2: My tendency is towards using immersive VR whenever possible.	[59]

**Table 2:** Reliability of individual indicators.

Outer Loadings (≥0.7)		Outer Loadings (≥0.7)	
Performance Expectancy (PE)		Facilitating Conditions (FC)	
PE1	0.866	FC1	0.876
PE2	0.740	FC2	0.718
PE3	0.841	FC3	0.842
Effort Expectancy (EE)		Behavioral Intention (BI)	
EE1	0.798	BI1	0.736
EE2	0.766	BI2	0.862
Social Influence (SI)		Use Behavior (UB)	
SI3	0.835	UB1	0.933
SI3	0.830	UB2	0.707
SI3	0.820		

Note. Individual Item reliability (>0.70).

**Table 3:** Construct reliability and convergent validity.

Constructs	Items	Loading (>0.7)	Average Variance Extracted (AVE) (≥0.5)	Composite Reliability (CR) (>0.70)
Performance Expectancy (PE)	PE-1	0.866	0.668	0.858
	PE-2	0.740		
	PE-3	0.841		
Effort Expectancy (EE)	EE-1	0.798	0.611	0.759
	EE-2	0.766		
Social Influence (SI)	SI-1	0.835	0.686	0.868
	SI-2	0.830		
	SI-3	0.820		
Facilitating Conditions (FC)	FC-1	0.876	0.664	0.855
	FC-2	0.718		
	FC-3	0.842		
Behavioral Intention (BI)	BI-1	0.736	0.643	0.782
	BI-2	0.862		
Use Behavior (UB)	UB-1	0.933	0.685	0.810
	UB-2	0.707		

Note. Individual item reliability (>0.70); composite reliability (>0.70); average variance extracted (≥0.50).

**Table 4:** Discriminant validity.

	PE	EE	SI	FC	BI	UB
PE	**0.818**					
EE	0.585	**0.782**				
SI	0.559	0.631	**0.828**			
FC	0.732	0.690	0.625	**0.815**		
BI	0.702	0.768	0.739	0.754	**0.802**	
UB	0.490	0.564	0.302	0.643	0.445	**0.828**

Note. The constructs in bold demonstrate successful discriminant validity (<0.85). PE = Performance Expectancy; EE = Effort Expectancy; SI = Social Influence; FC = Facilitating Conditions; BI = Behavior Intention; UB = Use Behavior.

**Table 5:** Path coefficients of the research hypotheses.

Hypotheses	Relationship	Std. Beta	Std. Error	T Values (>1.96)	*p* Values (<0.05)	Decision
$H_{1}$	PE -> BI	0.289	0.146	1.976	0.049	* Sig
$H_{2}$	EE -> BI	0.389	0.164	2.375	0.018	* Sig
$H_{3}$	SI -> BI	0.332	0.162	2.053	0.041	* Sig.
$H_{4}$	FC -> UB	0.714	0.249	2.870	0.004	* Sig.
$H_{5}$	BI -> UB	−0.093	0.225	0.415	0.679	Not Sig.

Note. PE = Performance Expectancy; EE = Effort Expectancy; SI = Social Influence; FC = Facilitating Conditions; BI = Behavioral Intention; UB = Use Behavior. * Relationships are significant at *p* < 0.05.

## Data Availability

Data will be made available on request.
